# A poroelastic immersed finite element framework for modelling cardiac perfusion and fluid–structure interaction

**DOI:** 10.1002/cnm.3446

**Published:** 2021-02-28

**Authors:** Scott I. Heath Richardson, Hao Gao, Jennifer Cox, Rob Janiczek, Boyce E. Griffith, Colin Berry, Xiaoyu Luo

**Affiliations:** 1 School of Mathematics and Statistics, University of Glasgow, Glasgow, UK; 2 GlaxoSmithKline plc, Stevenage, UK; 3 Departments of Mathematics, Applied Physical Sciences, and Biomedical Engineering, University of North Carolina, Chapel Hill, North Carolina, USA; 4 British Heart Foundation Glasgow Cardiovascular Research Centre, University of Glasgow, Glasgow, UK

**Keywords:** constitutive laws, Darcy flow, fibre-reinforced poroelastic material, fluid–structure interaction, heart perfusion, left ventricle, mixture

## Abstract

Modern approaches to modelling cardiac perfusion now commonly describe the myocardium using the framework of poroelasticity. Cardiac tissue can be described as a saturated porous medium composed of the pore fluid (blood) and the skeleton (myocytes and collagen scaffold). In previous studies fluid–structure interaction in the heart has been treated in a variety of ways, but in most cases, the myocardium is assumed to be a hyperelastic fibre-reinforced material. Conversely, models that treat the myocardium as a poroelastic material typically neglect interactions between the myocardium and intracardiac blood flow. This work presents a poroelastic immersed finite element framework to model left ventricular dynamics in a three-phase poroelastic system composed of the pore blood fluid, the skeleton, and the chamber fluid. We benchmark our approach by examining a pair of prototypical poroelastic formations using a simple cubic geometry considered in the prior work by Chapelle et al (2010). This cubic model also enables us to compare the differences between system behaviour when using isotropic and anisotropic material models for the skeleton. With this framework, we also simulate the poroelastic dynamics of a three-dimensional left ventricle, in which the myocardium is described by the Holzapfel–Ogden law. Results obtained using the poroelastic model are compared to those of a corresponding hyperelastic model studied previously. We find that the poroelastic LV behaves differently from the hyper-elastic LV model. For example, accounting for perfusion results in a smaller diastolic chamber volume, agreeing well with the well-known wall-stiffening effect under perfusion reported previously. Meanwhile differences in systolic function, such as fibre strain in the basal and middle ventricle, are found to be comparatively minor.

## 1 | INTRODUCTION

Maintaining cardiac pump function requires continuous perfusion of the cardiac muscle. Oxygenated blood is distributed via the coronary arteries and a complex network of capillaries to the working myocardium. Conditions that result in insufficient perfusion, such as stenosis in the large arteries, microvascular dysfunction or increased compression of the extracellular space, increase the likelihood of ischemic injury, infarction, and ultimately, death. Computational models of perfusion can help to understand both the damage caused by such occlusions and the impact of potential treatments. However, before analysing disruption within this system, it is important first to benchmark any model by examining an uninterrupted cardiac cycle. This involves, among other intricacies, the flow of blood across various scales and compartments as well as interactions with the myocardium.^1^

In our model, left ventricular dynamics involve interactions of three phrases, the solid, the pore fluid, and the ventricular blood flow within the chamber (the pericardium is neglected). We note that throughout this paper, the solid may be referred to as either the skeleton or the myocardium, the pore fluid is the coronary flow while the chamber flow is the cavity blood fluid. Techniques for explicitly modelling the coronary vascular network have been refined over several decades,^2–6^ but models accounting for interactions within the micro vascular network,^1^ where clinical perfusion is typically assessed,^7^ remain scarce. Further, despite continued improvement in the spatial resolution of clinical imaging, a fully detailed representation of the coronary vessels within the ventricular wall remains unachievable. However, obtaining this remains desirable due to the inherent link between myocardial perfusion and heart wall dynamics. Thus, to overcome this limitation, homogenised perfusion models, which do not account for the detailed network structure of the coronary vasculature, are often employed to study pressure and flow in the working myocardium. In such models Darcy’s law is sufficiently accurate so as to be used as a representation for blood flow.^8^ An example of such a model was developed by Hyde and Michler,^9,10^ who developed their multiscale myocardial perfusion model by parameterising compartments based on a discrete vascular network while simultaneously solving a static Darcy problem within each of these. This was subsequently coupled with a scalar transport model to quantify the behaviour of contrast agents during perfusion imaging.^11^

The myocardium contracts and relaxes within each heart beat and a static myocardial perfusion model will not capture the dynamics of the heart wall that will inevitably affect the perfusing blood within the myocardium. Several studies have incorporated the myocardial dynamics with the Darcy flow in the heart in a poroelastic framework.^8,12–14^ One of the earliest studies is from Huyghe et al,^12^ in which the myocardium was modelled as a spongy anistropic viscoelastic material in an axis-symmetric beating left ventricle. Later, Chapelle et al^8^ proposed a general poroelastic myocardial model that incorporated both the unsteady blood flow and a description of the myocardium as a nonlinear hyperelastic material. This study introduced several benchmark tests and ultimately simulated perfusion in the left ventricle. This model was shown to be capable of reproducing several key phenomena, including myocardial volume change resulted from perfusion. Subsequently, Cookson et al^13^ combined the multi-compartmental, static approach from Hyde et al^9^ and the poroelastic mechanical framework in Chapelle et al,^8^ as well as integrating clinical imaging into this modelling framework, and demonstrated that their final model proved capable of predicting the expected wall stiffening which occurs due to an increase of fluid mass. Most recently Lee et al^14^ coupled a poroelastic myocardial perfusion model to a one-dimensional representation of the coronary network and preformed detailed wave intensity analysis. Their results showed a strong consistency between their simulations and experimental observations.

A common aspect of previous modelling works on cardiac perfusion is the utilisation of the Lagrangian approach on both the porous media and the fluid constituents.^8,14^ However, despite its advantages in structure only simulations, the total Lagrangian approach poses difficulties when coupled to ventricular cavity blood flow due to the large deformation of the heart walls and valves.^15^ The immersed finite element (IFE) method, combines the strength of the immersed boundary (IB) methods^16^ with an immersed finite element methods for the elastic structure, enables effective modelling of fluid–structure interaction (FSI) with large structural deformation. IFE further avoids the use of body-conforming discretizations between the fluid and structure,^17^ and hence overcomes mesh distortion issues in body-fitted approaches.^18,19^ One such an example is shown in simulations of FSI in the vicinity of the cardiac valves.^20,21^

The extension of IFE formulations to treat poroelastic structures is relatively recent.^22,23^ Strychalski et al^22^ developed a poroelastic IB method for cellular mechanics, using a Lagrangian reference frame while the fluid moving both in and outside of the cell is described in Eulerian form although this approach only considers the dilute limit. However, this approach resulted in simulations which successfully reproduced cellular blebbing and crawling. Rauch et al^23^ investigated how cells migrate in a three dimensional porous extracellular matrix by developing a finite element based immersed method. In that approach, the cellular matrix is modelled as two distinct constituents, with one represented as a nearly incompressible fluid and the other an incompressible Darcy-Brinkman flow. This model also accounts for the effects of contacts that occur due to cell migration. These studies demonstrate how various mechanical interactions can be readily incorporated into a poroelastic IB framework. More recently, a consistent approach for fluid–structure-contact interaction based on a porous flow model for rough surface contact and an approach for vascular tumour growth based on a hybrid embedded/homogenised treatment of the vasculature within a multiphase porous medium model was developed by Wall and coworkers.^24,25^ However, these models are complex and have not been used to model the myocardium.

This study extends our immersed finite element framework,^19,26^ which was introduced to describe incompressible elastic structures immersed in a viscous incompressible fluid, to treat poroelastic immersed structures, and it applies this poroelastic immersed formulation to simulate cardiac perfusion and FSI. We specifically incorporate a three phase poroelastic cardiac modelling approach, an important consideration when investigating, for example, effective perfusion of the heart during heart diseases such as myocardial infarction.^1,3^

## 2 | POROELASTIC MODEL FORMULATION

This section introduces the poroelastic model for the myocardium and the corresponding hyperelastic constitutive law. In the immersed formulation, the porous myocardium is immersed in a fixed fluid domain using an immersed finite element framework as detailed in Section 3. This formulation uses both Eulerian and Lagrangian frames. Fixed Eulerian are **x** = (*x*_1_, *x*_2_, *x*_3_), and Lagrangian (material) coordinates attached to the structure are **X** = (*X*_1_, *X*_2_, *X*_3_).

### 2.1 | Porous medium

**FIGURE 1:**
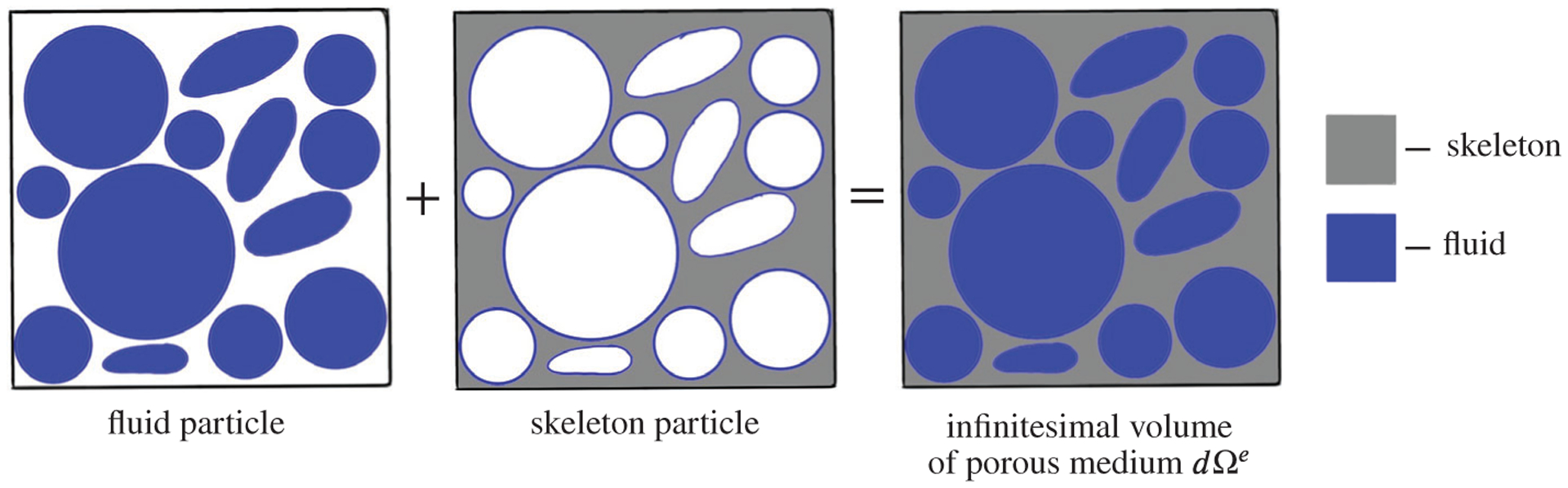
The representative elementary volume of the elastic porous medium *d*Ω^*e*^, which contains both fluid and solid phases

We model myocardial tissue as a saturated porous medium composed of two constituents: the perfusate, that is, the blood, and the solid (skeleton), that is, myocytes and collagen scaffold. We assume that the pores are distributed in a representative element volume of the porous medium, a rough illustration of which is shown in Figure 1 (although we note this is not a particular distribution). The homogenised porous medium then consists of two overlapping averaged phases of fluid and solid.^27^ For clarity, the quantities that are used to describe the two macroscopic phases can be expressed as the fluid, *ϕϑ*^*f*^, and the solid (or the skeleton), (1 − *ϕ*)*ϑ*^*s*^, in which *ϑ* can indicate density, velocity, stress, pressure, or strain, and superscripts *f* or *s* denote fluid or skeleton, respectively. Hence, the corresponding quantities of the porous medium (also known as the mixture) are then expressed as *ϕϑ*^*f*^ + (1 − *ϕ*)*ϑ*^*s*^, so that *ϕ* = 1 corresponds to an entirely fluid material region, and *ϕ* = 0 corresponds to one that is only solid.

### 2.2 | Skeleton

Let ***χ***(**X**, *t*) denote the physical position of material point **X** at time *t*. The physical region occupied by the skeleton at time *t* is $\chi \left(\Omega_{0}^{s},t\right)$ (where $\Omega_{0}^{s}$ indicates the material co-ordinate domain) and the deformation gradient associated with the skeleton is $F=\frac{\partial \chi }{\partial X}$. The material derivative associated with the solid phase of J(X,t)=detF is

(1) $$\frac{D}{Dt}J(X,t)=J(X,t)\nabla_{x}\cdot v^{s}(x,t),$$

where $v^{\text{s}}(x,t)=\frac{\partial \chi (X,t)}{\partial t}$ is the velocity of the skeleton. Additionally, conservation of mass in the skeleton takes the form,

(2) $$J(X,t)\rho^{s}(1-\phi (\chi (X,t),t))=\rho_{0}^{s}\left(1-\phi_{0}\right),$$

where *ρ*^*s*^ is density of the solid and *ρ*_0_, *ϕ*_0_ are respectively the initial density and fluid volume per unit tissue volume.

### 2.3 | Pore fluid

The transport of fluid through porous medium can be modelled by Darcy’s law. In Eulerian form, this is

(3) w(x,t)=−K∇p(x,t),

where **K** is the permeability tensor and *p* is the pore pressure. The perfusion (or Darcy) velocity **w**(**x**, *t*) is

(4) $$w(x,t)=\phi \left(v^{\text{f}}(x,t)-v^{s}(x,t)\right),$$

where **v**^f^(**x**, *t*) is velocity of the pore fluid. The mass conservation of the fluid phase is then

(5) $$\frac{\partial }{\partial t}\left(\phi (x,t)\rho^{f}\right)+\nabla \cdot \left(\rho^{f}\phi (x,t)v^{\text{f}}(x,t)\right)=\rho^{f}s(x,t),$$

where *s*(**x**, *t*) is a sink/source per unit volume. After substituting (4) into (5), we can write

(6) $$\frac{D}{Dt}\left(\rho^{f}\phi (x,t)\right)+\rho^{f}\phi (x,t)\nabla \cdot v^{\text{s}}(x,t)=\rho^{f}s(x,t)-\nabla \cdot \left(\rho^{f}w(x,t)\right),$$

Let *M*(**X**, *t*) be the added fluid mass,

(7) $$M(X,t)=\rho^{f}J(X,t)\phi ((\chi (X,t),t)-\rho_{0}^{f}\phi_{0},$$

so that

(8) $$\frac{D}{Dt}M(X,t)=J(X,t)\frac{D}{Dt}(\rho^{f}\phi ((\chi (X,t),t))+\rho^{f}\phi (\left(\chi (X,t),t)\frac{D}{Dt}J(X,t\right).$$

Using (1), Equation (8) can be rewritten as

(9) $$J(X,t)\frac{D}{Dt}(\rho^{f}\phi ((\chi (X,t),t))+J(X,t)\rho^{f}\phi (\left(\chi (X,t),t)\nabla_{x}\cdot v^{\text{s}}(x,t)=\frac{D}{Dt}M(X,t\right).$$

Comparing Equations (9) and (6) give

(10) $$\frac{1}{J(X,t)}\frac{D}{Dt}M(X,t)+\nabla_{x}\cdot \left(\rho^{f}w(x,t)\right)=\rho^{f}s((\chi (X,t),t),$$

which agrees with the expression used by Chapelle et al.^8^

### 2.4 | Momentum equation for the porous medium

The momentum equation for the porous medium is given by

(11) $$\rho^{s}(1-\phi (x,t))a^{s}(x,t)+\rho^{f}\phi (x,t)a^{f}(x,t)=\nabla \cdot \sigma^{e}(x,t),$$

where **a**^*f*^(**x**, *t*), **a**^*s*^(**x**, *t*) are the acceleration vectors of the fluid and solid, respectively, and ***σ***^*e*^(**x**, *t*) denotes the Cauchy stress tensor of the mixture. If we neglect the discrepancy between the fluid and solid accelerations, as in Chapelle et al,^8^ then (11) simplifies to

(12) $$\frac{m(x,t)+\rho_{0}}{J(X,t)}\frac{D}{Dt}v^{s}(x,t)=\nabla \cdot \sigma^{e}(x,t),$$

where $\rho_{0}=\phi_{0}\rho_{0}^{f}+\left(1-\phi_{0}\right)\rho_{0}^{s}$, $m(x,t)=\rho^{f}J\phi -\rho_{0}^{f}\phi_{0}$ and we have made use of (7), (11) while also assuming that **a**^*s*^ = *D***v**^s^/*Dt*.

### 2.5 | Constitutive laws for porous medium

For an isothermal poroelastic material, the free energy Ψ per unit volume in the reference configuration depends only on F and *m*^27^, meaning that Ψ=Ψ(F,m), so that

(13) $$\dot{\Psi }=\text{tr}\left(\frac{\partial \Psi }{\partial F}\dot{F}\right)+\frac{\partial \Psi }{\partial m}\dot{m}.$$

Using (13), as well as the formula $\dot{F}=LF$, where L is the spatial velocity gradient of the skeleton, the entropy production inequality is^28,29^

(14) $$\text{tr}\left[\left(JF\frac{\partial \Psi }{\partial F}-\sigma \right)L\right]+\left(\frac{\partial \Psi }{\partial m}-g_{m}\right)\dot{m}>0.$$

The inequality (14) must hold for arbitrary L and *ṁ*, and cannot do so unless

(15) $$\sigma =\frac{F}{J}\frac{\partial \Psi }{\partial F}\text{ and }g_{m}=\frac{\partial \Psi }{\partial m},$$

where *g*_*m*_(*p*) is the free enthalpy (or Gibb’s free energy). This characterises the pore fluid constitutive behaviour through

(16) $$\frac{1}{\rho_{0}^{f}}=\frac{\partial g_{m}}{\partial p},\text{ or }p-p_{0}=\rho_{0}^{f}\frac{\partial \Psi }{\partial m},$$

where *p*_0_ defines pore pressure in the reference state. Equations (13)–(16) set the requirements of the constitutive relations for the poroelastic medium.

Herein, we employ a free energy function in a form that is similar to that used by Chapelle et al^28^

(17) $$\Psi =\Psi^{\text{s}}(F,m)+m\Psi^{m}\left(\frac{1}{\rho_{0}^{f}}\right),$$

where Ψ^s^ is the free energy of the skeleton (per unit volume of the reference configuration) and $\Psi^{m}=g_{m}-p/\rho_{0}^{f}$ is the Helmholtz free energy per unit mass. Following Coussy and Uhm,^28^ we use the splitting

(18) $$\Psi^{s}(F,m(x,t))=W^{\text{hyp}}(F)+W^{\text{bulk}}(J(X,t),m(X,t)),$$

where *W*^hyp^ is the deviatoric potential representing the constitutive behaviour of the skeleton as a structure, and *W*^bulk^ describes how the energy depends on the solid phase volume changes (since *m*(**x**, *t*) is proportional to *J*(**X**, *t*)*ϕ*(**x**, *t*)). In addition, we choose *m*(**x**, *t*)Ψ^*m*^ via,^8^

(19) $$m(x,t)\Psi^{m}\left(\frac{1}{\rho_{f0}}\right)=M_{b}b\frac{m(x,t)}{\rho_{0}^{f}}(J(X,t)-1)f(J(X,t))+\frac{1}{2}M_{b}{\left(\frac{m(x,t)}{\rho_{0}^{f}}\right)}^{2}f(J(X,t))-\kappa_{0}\text{ln}\left(\frac{m(x,t)}{\rho_{0}^{f}}+\phi_{0}\right),$$

where *M*_*b*_, *b*, and *κ*_0_ are constants respectively defining the Biot modulus, a parameter characteristic of the skeleton, and a penalty term to ensure that 0 < *ϕ* < 1. In addition we use^28^

(20) $$f(J(X,t))=\frac{2(J(X,t)-1-\text{ln}(J(X,t)))}{{\left(J(X,t)-1\right)}^{2}}.$$

Differentiating Equation (19) with respect to *m* and substituting into (16) yields the interstitial pore pressure,

(21) $$p(x,t)-p_{0}=\rho_{0}^{f}\frac{\partial \Psi^{m}}{\partial m}=M_{b}\left(b(1-J(X,t))+\frac{m(x,t)}{\rho_{0}^{f}}\right)f(J(X,t))-\kappa_{0}\frac{\rho_{0}^{f}}{m(x,t)+\rho_{0}^{f}\phi_{0}}.$$

Note that on the left hand side of Equation (21), *p*_0_ = *κ*_0_*ϕ*_0_, which ensures that *m* = 0, *p* = 0, and *J* = 1 form a trivial solution.

## 3 | IMMERSED FORMULATION OF THREE-PHASE FLUID-POROELASTIC STRUCTURE INTERACTION

### 3.1 | Immersed poroelastic structures

**FIGURE 2:**
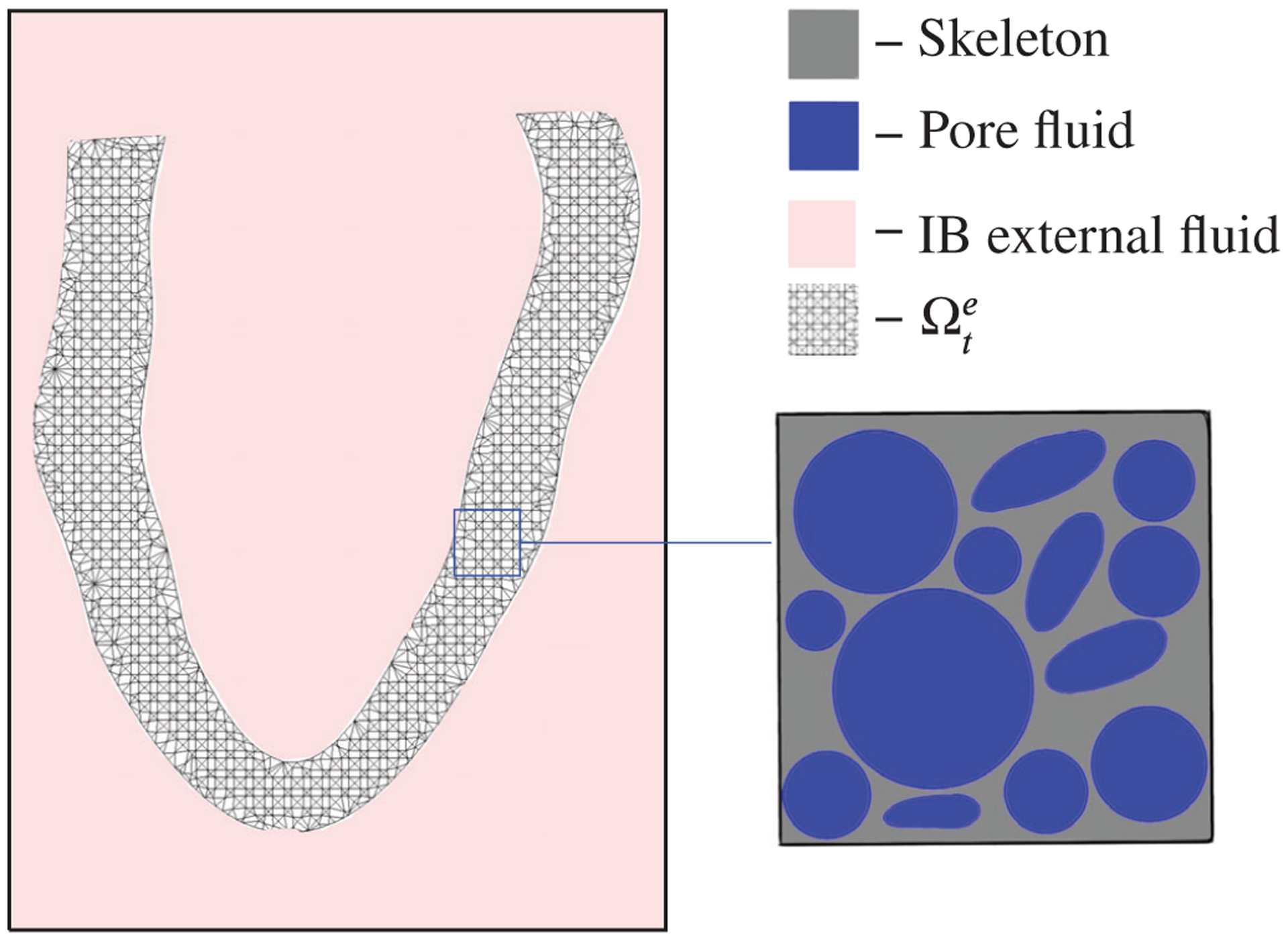
The three-phase fluid–structure interaction domain, wherein the poroelastic structure is bathed in a viscous incompressible fluid that is distinct from the pore fluid. Different phases are shown in pink (exterior fluid), grey (solid), and pore fluid (blue)

We model fluid-poroelastic structure interaction in the particular case that the poroelastic medium is bathed in a viscous incompressible fluid. We assume that the exterior fluid in which the structure is immersed is distinct from the interior pore fluid within the poroelastic medium. We define this surrounding fluid as the bathing fluid while noting that the exterior fluid occupies the region $\Omega_{t}^{\text{f}}$ at time *t*, and the poroelastic structure occupies the region $\Omega_{t}^{\text{e}}$. We assume that these regions are non-overlapping and, further, that they form a fixed computational domain $\Omega =\Omega_{t}^{\text{f}}\cup \Omega_{t}^{\text{e}}$ (Figure 2).

We assume that density is constant across all constituents, that is, $\rho =\rho_{0}=\rho^{s}=\rho^{f}=\rho_{0}^{s}=\rho_{0}^{f}=\rho_{0}^{\text{exterior fluid}}$. This allows Equations (2) and (7) to be combined so that

(22) $$J(X,t)=1+\frac{M(X,t)}{\rho },$$

and

(23) $$\frac{D}{Dt}M(X,t)=\rho \frac{D}{Dt}J(X,t).$$

Since (1) and (10) are alternative formulations for *Dm*/*Dt* and *DJ*/*Dt*, after minimal algebra the following expression can be derived by substituting these into (23)

(24) $$\nabla \cdot v^{s}(x,t)=s(x,t)-\nabla \cdot w(x,t).$$

### 3.2 | Continuous formulation

In typical immersed formulations of fluid–structure interaction, a common material velocity field **v**(**x**, *t*) is used for both the structure and the (exterior) fluid. To develop such a formulation for a poroelastic immersed structure, we track the velocity field of the skeleton, so that the deformation and velocity fields of the skeleton can be readily available for the Darcy flow model. The velocity of the IFE system, defined as

$$v(x,t)=(\begin{array}{ll} v^{\text{s}}(x,t), & \chi (x,t)\in \Omega_{t}^{\text{e}},\\ v_{\text{ib}}^{\text{f}}(x,t), & \text{otherwise,} \end{array}$$

where $v_{\text{ib}}^{\text{f}}$ defines the Eulerian velocity field in $\Omega_{\text{ib}}^{f}$ which is the bathing fluid. The momentum equation for the three-phase system is

(25) $$\rho \frac{Dv(x,t)}{Dt}=\nabla \cdot \sigma ,$$

and the region specific Cauchy stress tensor is

(26) $$\sigma (x,t)=(\begin{array}{ll} p_{\text{ib}}(x,t)I+\mu \left(\nabla v(x,t)+{\left(\nabla v(x,t)\right)}^{T}\right)+\sigma^{e}(x,t), & x\in \Omega_{t}^{\text{e}},\\ p_{\text{ib}}(x,t)I+\mu \left(\nabla v(x,t)+{\left(\nabla v(x,t)\right)}^{T}\right), & \text{otherwise}, \end{array}$$

where ***σ***^*e*^(**x**, *t*) is the elastic Cauchy stress tensor of the mixture, *p*_ib_(**x**, *t*) + *μ*(∇**v**(**x**, *t*) + (∇**v**(**x**, *t*))^*T*^) is the fluid-like stress tensor, *μ* is the viscosity of the bathing fluid, and *p*_ib_ is the Lagrange multiplier to ensure the continuity equation is satisfied. The continuity equation for the poroelastic IB/FE system is given by

(27) $$\nabla \cdot v^{s}(x,t)=s^{*}(x,t)=s(x,t)-\nabla \cdot w(x,t),$$

which is the same as (24).

Along with the momentum Equation (25), we use the Lagrangian form of the Darcy system to determine the dynamics of the pore fluid:

(28) $$\frac{\partial M(X,t)}{\partial t}+\nabla_{X}\cdot (\rho W(X,t))=\rho S(X,t),$$

(29) $$FW(X,t)=-J(X,t)KF^{-T}\nabla_{\text{X}}p(x,t),$$

(30) $$p(x,t)-p_{0}=\rho \frac{\partial \Psi^{m}}{\partial m}.$$

The remaining dynamic equations are^19^

(31) $$\rho^{*}\frac{Dv(x,t)}{Dt}=\nabla \cdot piI+\mu \nabla^{2}u+f^{e},$$

(32) $$\nabla \cdot v(x,t)=s^{*}(x,t),$$

(33) $$f^{e}=\int_{\Omega_{0}^{e}}F^{e}(X,t)\delta (x-\chi (X,t))dX,$$

(34) $$\int_{\Omega_{0}^{s}}F^{e}(X,t)\cdot V(X)dX=-\int_{\Omega_{0}^{s}}{\mathbb{P}}^{e}(X,t):\nabla_{\text{x}}V(X)dX,$$

(35) $$\frac{\partial \chi (X,t)}{\partial t}=\int_{\Omega }v(x,t)\delta (\chi (X,t)-x)dx,$$

where *δ*(**x**) is the Dirac delta function and ${\mathbb{P}}^{e}(X,t)$ is the first Piola-Kirchhoff stress tensor for the elastic constituents, which can be computed via ${\mathbb{P}}^{e}(X,t)=\frac{\partial W}{\partial F}=J(x,t)\sigma F^{-T}$ and (15). Finally the fluid and structural constituents are connected via

(36) $$S^{*}(X,t)=S(X,t)-\nabla_{X}\cdot W(X,t),\;s^{*}(x,t)=\int_{\Omega_{0}^{s}}S^{*}(X,t)\delta (x-\chi (X,t))dX.$$

### 3.3 | Discrete formulation

Numerically the two systems summarised above are updated in sequence. Equations (31)–(35) are first used to find **v**^*n* + 1^, $p_{ib}^{n+1}$, *χ* ^*n* + 1^, $F^{n+1}$ and *J*^*n*+ 1^ (note that the superscript *n* defines the value at the current time step and *n* + 1 the following time step) with the updated F and *J* then used in Equations (28)–(30) to find *m*^*n*+1^, **W**^*n*+1^ and *p*^*n*+1^. The new Darcy velocity, **W**^*n*+1^, is then passed, along with *S*, back to the IB/FE system via Equation (36).

To update Equations (28)–(30) a split step temporal scheme is employed where the primary variable *p* is updated implicitly. This allows appropriate boundary conditions to be enforced on the pore pressure while **W** and *m* can thereafter be recovered through simple explicit schemes, found by reformulating Equations (28)–(30).

To achieve an appropriate form for updating *p*, Equation (29) is substituted into (28) while (30) is first differentiated with respect to time, and thereafter, used to obtain a relationship between *∂m*/*∂t* and *∂p*/*∂t*. Differentiating Equation (30) gives

(37) $$\frac{\partial p}{\partial t}=\rho \frac{\partial }{\partial t}\left(\frac{\partial \Psi^{m}}{\partial m}\right)=A(J)\frac{\partial m}{\partial t}+B(J)\frac{\partial J}{\partial t}+C(m,J)-D(m),$$

where

$$A(J)=\frac{M_{b}f(J)}{\rho },\;B(J)=-M_{b}f(J)b,\;C(m,J)=M_{b}f^{\prime }(J)\left(b(1-J)+\frac{m}{\rho }\right),\;D(m)=\frac{\partial }{\partial t}\left(\frac{\kappa_{0}\rho }{m+\rho \phi_{0}}\right).$$

As indicated, Equation (37) is re-written in terms of *∂m*/*∂t* before being substituted into (28) so that, after also using Equation (29), we obtain

(38) $$\frac{1}{\rho }\frac{\partial p}{\partial t}-\rho JA\nabla_{X}\cdot \left(F^{-1}KF^{-T}\nabla_{X}p\right)=\rho SA+B\frac{\partial J}{\partial t}+C=E(m,J).$$

As indicated above when updating the Darcy system, the primary variables (*p*^*n*^, **W**^*n*^, *m*^*n*^) are first advanced to a half way temporal time step ($p^{n+\frac{1}{2}}$, $W^{n+\frac{1}{2}}m^{n+\frac{1}{2}}$) before solving for (*p*^*n*+1^, **W**^*n*+1^, *m*^*n*+1^). To preform the first step of finding *p*^*n*+1/2^, Equation (38) is discretised so that

(39) $$\frac{1}{\rho }\frac{p^{n+1/2}-p^{n}}{\Delta t/2}-\rho J^{n}A\nabla_{X}\cdot \left(F^{-1}KF^{-T}\nabla_{X}p^{n+1/2}\right)=E\left(m^{n},J^{n}\right),$$

with all *p*^*n* + 1/2^ terms gathered on the left before solving for this implicitly. This is carried out using a standard Galerkin finite element discretization in space with first-order Lagrangian basis functions. Next, **W**^*n* + 1/2^ is obtained through a simple reformulation of Equation (29)

(40) $$W^{n+1/2}=-J^{n}F^{-1}KF^{-T}\nabla_{X}p^{n+1/2},$$

and finally by substituting Ψ^m^ into Equation (30), *m*^*i* + 1/2^ is obtained via we have

(41) $$m^{n+1/2}=\rho_{0}^{f}\left(\frac{1}{M}\left(p^{n+1/2}-p_{0}+C\left(m^{n},J^{n}\right)+B\left(J^{n}\right)\left(J^{n}-1\right)\right)\right).$$

Equations (39)–(41) conclude the update to the intermediate variables (*p*^*n* + 1/2^, **W**^*n* + 1/2^, *m*^*n* + 1/2^). The second updates to *p*^*n* + 1^ and **W**^*n* + 1^ are identical to those in (39) and (40), while *m*^*n* + 1^ is now found by Equation (28), leading to

(42) $$\frac{m^{n+1}-m^{n+1/2}}{\Delta t/2}=\rho S^{n+1}-\nabla_{X}\cdot \left(\rho W^{n}\right).$$

A schematic illustration of the overall algorithm is provided in the Appendix.

## 4 | RESULTS

In this section, we first present a pair of verification tests on a cube, comparing against published results from Chapelle et al,^8^ before extending this example by retaining the geometry but using an orthotropic constitutive law that has been widely used in the cardiac modelling literature. Finally, we apply this poroelastic IB/FE framework to a subject-specific human left ventricle (LV).

In these simulations, all three primary variables in the Darcy system are initialised to 0, and boundary conditions are enforced during the implicit update of the pore pressure, *p*. The solver for the Darcy system and IBAMR software run independently from each other although they are coupled with several variables being passed between these at each time step.

All simulations are performed at the School of Mathematics and Statistics at the University of Glasgow.

### 4.1 | Verification tests

To verify our poroelastic IB/FE framework with the results from Chapelle et al^8^ the free energy is formulated similarly, that is

(43) $$\Psi^{s}=W^{\text{hyp}}+W^{\text{bulk}},$$

but make necessary adjustments in the *W*^bulk^ term to ensure that incompressibility of the skeleton is enforced, that is, $J=1+\frac{m}{\rho_{0}^{f}}$. Thus the individual components of (43) are defined as

(44) $$W^{\text{hyp}}=\kappa_{1}\left(I_{1}-3\right)+\kappa_{2}\left(I_{2}-3\right),\;W^{\text{bulk}}=K_{s}{\text{ln}}^{2}\left(J-\frac{m}{\rho_{0}^{f}}\right),$$

where *κ*_1_, *κ*_2_, and *K*_*s*_ are material parameters, *I*_1_ = trace(**C**), and $I_{2}=\frac{1}{2}\left({\left(\text{tr}(C)\right)}^{2}-\text{tr}\left(C^{2}\right)\right)$ where $C=F^{T}F$. When comparing to the results of Chapelle et al,^8^ we note that the penalty term in (44) approximately imposes mass conservation on the skeleton, while the comparable term used previously^8^ approximately imposes *J* = 1. In fact, we do not wish to impose *J* = 1 because the porous medium is compressible as a result of the added mass from the Darcy flow, although both the skeleton and blood are individually incompressible. Thus in (44), we ensure that the mass of the skeleton shall be conserved, that is $J=1+\frac{m}{\rho_{0}^{f}}$. In the limiting case of the porosity being zero then *J* = 1 will be enforced, which represents a purely hyperelastic material. It is worth mentioning that after solving the continuity equation in the IB/FE system, *p*_ib_ also acts as a Lagrange multiplier to enforce ∇ · **v** = *s*, and it is an exact Lagrange multiplier for that constraint. The study of Vadala et al^26^ demonstrates the importance of including such volumetric energies in immersed formulations; see also our prior work on immersed models of ventricular mechanics.^30^

As in Chapelle et al,^8^ we assume isotropic permeability, that is, ***K*** = *k****I***, and we do not change the value for any parameters or boundary conditions except for *β*_*v*_, which was corrected for the drainage case (following private communication with the authors). Table 1 lists all the parameters used in our simulations. The cube is immersed into a computational domain of size 1 × 1 × 1 cm^3^ and discretised using a 96 × 96 × 96 Cartesian grid.

#### 4.1.1 | Swelling for a cube

**FIGURE 3:**
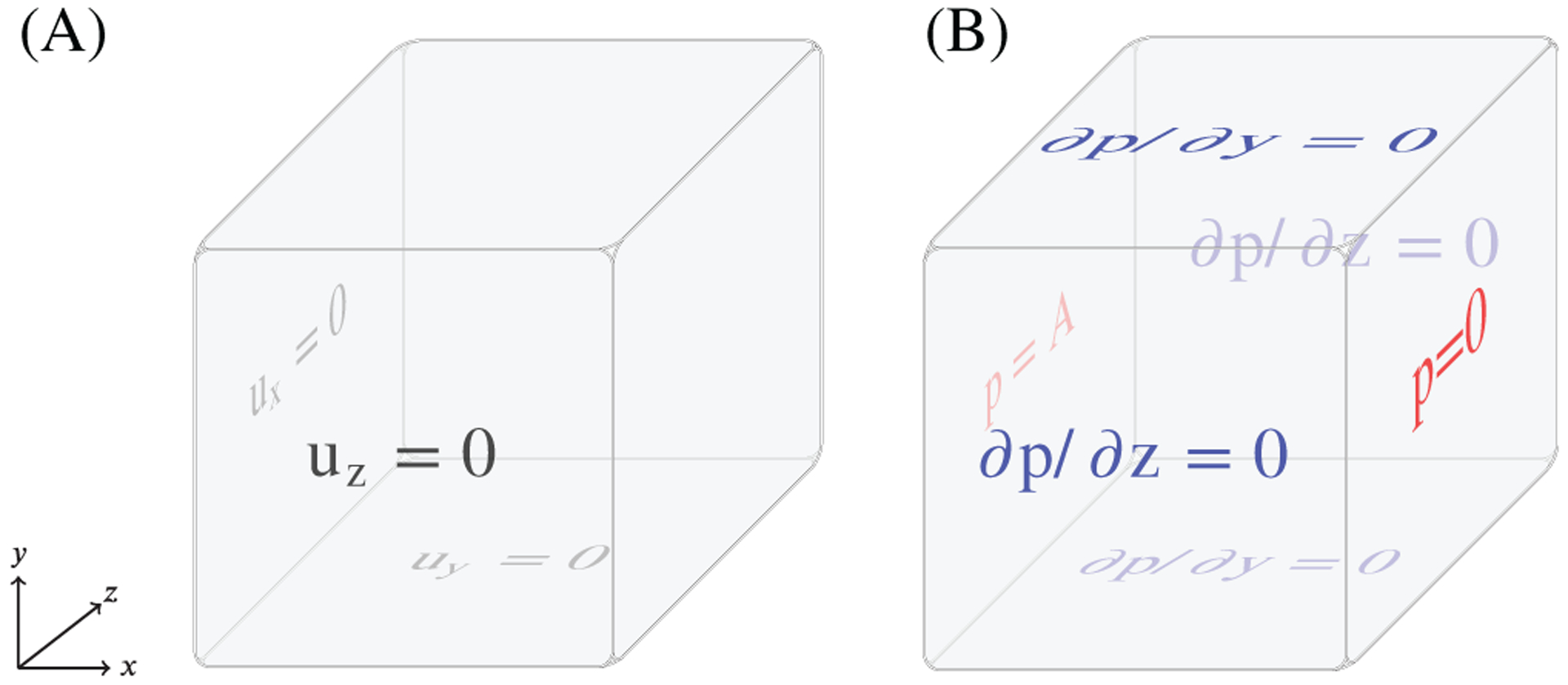
The boundary conditions which are enforced on the solid, (A), and fluid, (B), in the swelling test (as in Chapelle et al^8^). Note that in the boundary conditions for the fluid, *A* = 10^3^(1 − exp (−*t*^2^/0.25))Pa and *u*_*x*_, *u*_*y*_, *u*_*z*_ refer to the respective displacement components

In the swelling test, zero pressure is applied to the outer boundary of the computational domain. To keep the immersed porous medium in place, normal displacements are set to be zero for the planes *x* = 0, *y* = 0, and *z* = 0. There is no sink or source of pore fluid, that is, *S* = 0. A time-dependent pressure, *p*_bc_ = 10^3^(1 − exp(−*t*^2^/0.25)), is applied to the face *x* = 0, increasing from 0 to 1 kPa, while *p* is kept zero at the face *x* = 1. Zero-flux boundary conditions are applied to the other four faces. These boundary conditions are summarised in Figure 3.

To determine an appropriate grid size for all forthcoming simulations, a grid convergence test is carried for this swelling cube. Five different grid sizes are chosen, including 64^3^, 80^3^, 96^3^, 112^3^, and 128^3^. The pore pressure at the cube centre and the maximum displacements of the skeleton are compared with the values from the mesh with 128^3^, as shown in Table 2. Notice that the differences of the mesh with 96^3^ are within 5% of those obtained using a 128^3^ grid. Therefore, for the sake of computational efficiency, we use a 96^3^ grid for all subsequent computations.

**FIGURE 4:**
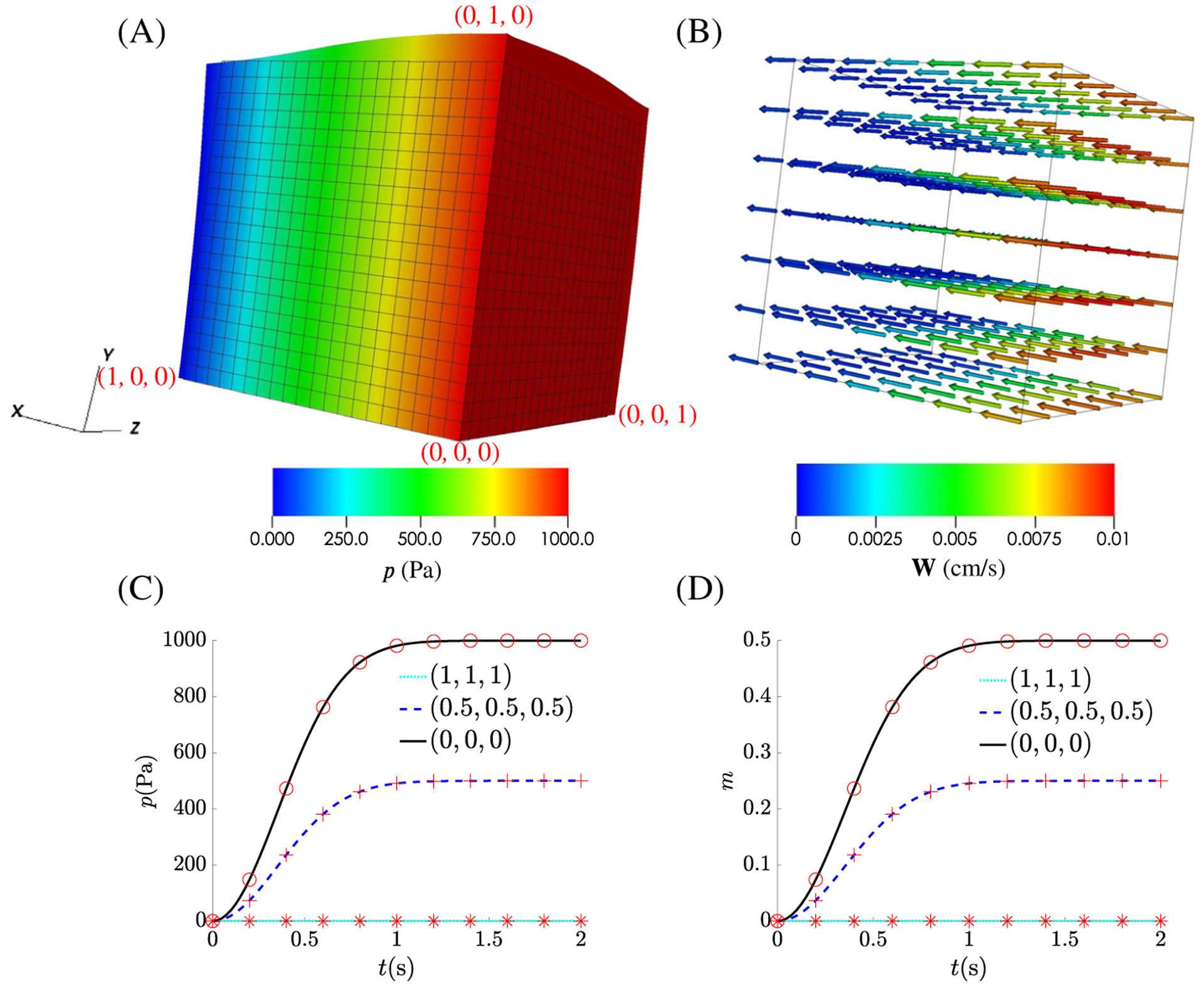
Results from the Swelling test: (A) pore pressure p; (B) Darcy velocity **W**; (C) pore pressure, p, as a function of time at three points within the cube fixed in the current configuration (bottom left, centre and top right); (D) increased mass over time at these same points. The results from Chapelle et al^8^ are plotted as an insert to each graph for easy comparison while 10 points have also been picked from these graphs and overlayed as red stars, crosses and circles on our results. So as to make the respective planes clear in (A) the point (*x*, *y*, *z*) = (0,0,0) is indicated along with three others

The results of the swelling test at steady state when the inlet pressure reaches its limits is shown in Figure 4. The cube swells like a sponge because of the increased pore pressure near the face *x* = 0, with the swelling gradually tapering towards the face *x* = 1, as shown in Figure 4(A). Figure 4(B) shows the Darcy velocity field **w**, and it is clear that the pore fluid mainly flows along the *x*-axis. Figure 4(C) shows the time profile of the added mass *m* at three different points: (0, 0, 0); (0.5, 0.5, 0.5); and (1, 1, 1), and the corresponding pore pressure profiles are shown in Figure 4(*d*). By comparing these results to those of Chapelle et al^8^ in Figure 4(C, D), we can see the two formulations produce nearly identical results.

#### 4.1.2 | Drainage for a cube

In the drainage test, the pressure in outer boundaries of the fluid domain is zero, the skeleton is fixed at (0,0,0), and zero normal displacements are applied on the planes *x* = 0, *y* = 0 and *z* = 0. An external force on the solid of 10 kPa is gradually applied on all the faces of the cube with *P*_*v*_ = 10^4^(1 − exp(−*t*^2^/0.04)) Pa. For the pore flow, no-flux condition is applied to all the faces, but the pore fluid is connected to a pressure sink term which is distributed throughout the skeleton. This is defined as *S* = − *β*(*p* − *p*_sink_) with *β* = 10^−4^s^−1^Pa^−1^ and *p*_sink_ = 0 Pa, which allows for volume change within the poroelastic structure.

**FIGURE 5:**
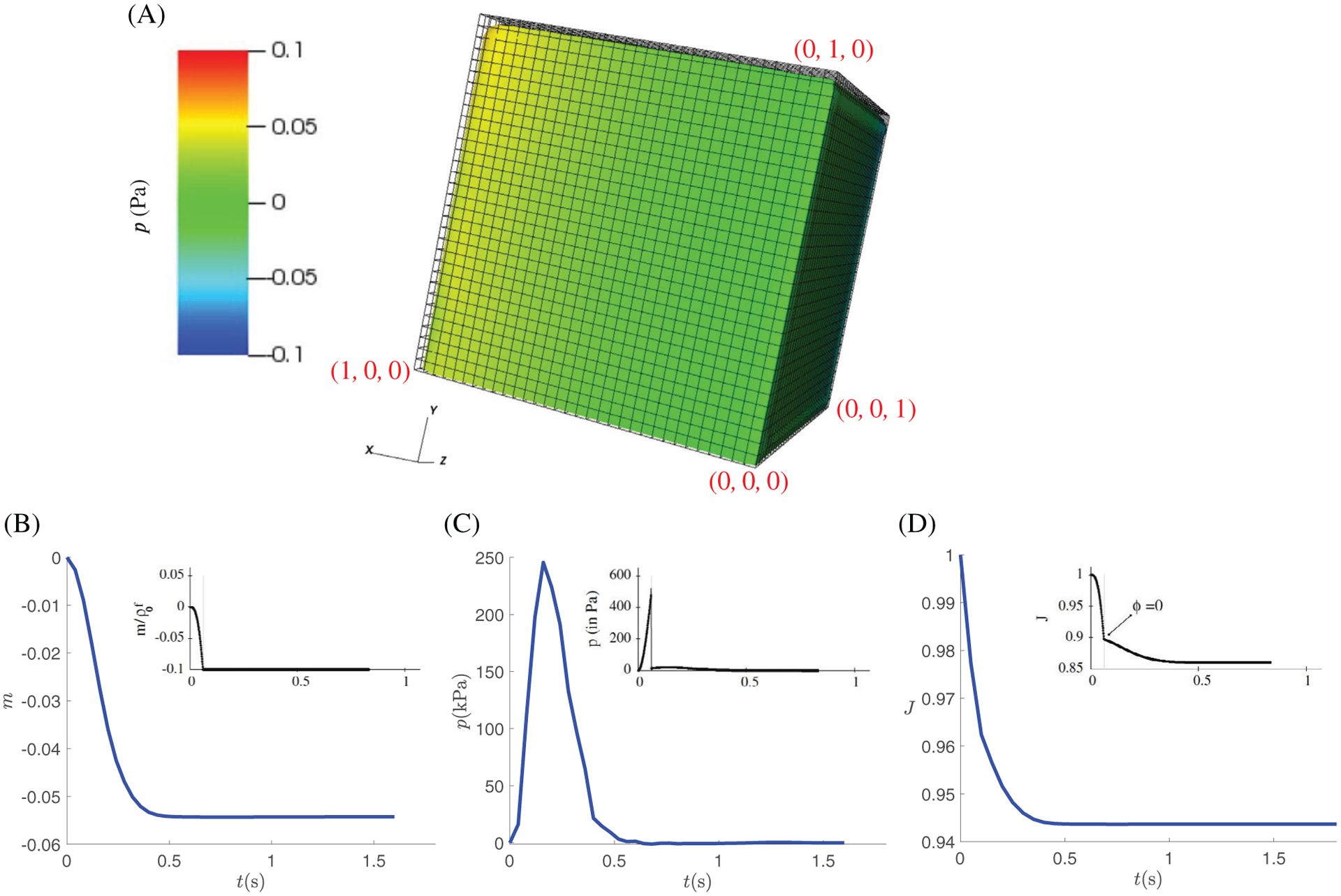
Drainage test results, (A) drainage of the cube from the original position (shown as mesh) upon an external pressure, and the time history of the volume averaged values of (B) added mass, (C) pore pressure, and (D) Jacobian *J*. The respective graphs from Chapelle et al are included alongside the results from our simulations. We note that while the profiles are similar the graphs approach different steady states, due to running with different values of some constants, as discussed in the text. So as to make the respective planes clear in (A) the point (*x*, *y*, *z*) = (0,0,0) is indicated along with three others

Figure 5 shows the drainage test results, in which the pore fluid is connected to a pressure sink, which drains the pore fluid out and shrinks the cube. Our results again show good agreement with the results from Chapelle’s study for *p*, *m* and *J*, in particular their patterns. However, we note that the values are not identical as in the swelling case. For instance, the sharp drop in the pore pressure which is seen in the results of Chapelle et al is no longer present due to differences between the three-phases immersed boundary approximation and a purely finite element approximation. Other reasons include: (1) one parameter relating to the sink/source (*β*_*v*_) was adjusted, after communicating with the authors, to ensure that it was sufficiently large enough to provide the pore pressure drop which is reported in fig. 2 of Chapelle et al^8^; (2) the mass conservation constraint is different compared to the work of Chapelle et al, in which *J* = 1 was imposed, instead we impose *J* = 1 − *m*/*ρ*_*f*_, see Equation (44) for details. Presumably, the mass conservation constraint will largely affect the levels of *J* and *m* and *p*, as we see in Figure 5.

### 4.2 | Fibre-reinforced material models

In Chapelle et al,^8^ the material property of the skeleton is considered to be nonlinear but isotropic. However, it is now known eperimentally that the mechanical property of the myocardium is nonlinear and anisotropic. Therefore, we use the Holzapfel–Ogden (HO) strain energy function^31^

(45) $$\begin{array}{ll} W^{\text{hyp}} & =\frac{a}{2b}\text{exp}\left[b\left(I_{1}-3\right)\right]+\sum_{\text{i}=\text{f},\text{s}}\frac{a_{i}}{2b_{i}}\left\{\text{exp}\left[b_{i}I_{4i}^{⋆2}\right]-1\right\}+\frac{a_{\text{fs}}}{2b_{\text{fs}}}\left\{\text{exp}\left[b_{\text{fs}}I_{8\text{fs}}^{2}\right]-1\right\},\\ W^{\text{bulk}} & =K{\left(\text{ln}(J-m)\right)}^{2}, \end{array}$$

where the material constants *a*, *a*_f_, *a*_s_, *a*_fs_, *b*, *b*_f_, *b*_s_, *b*_fs_, and $I_{4i}^{⋆}=\text{max}\left(I_{4i},1\right)-1$, *I*_4f_ = **f**_0_ · (**Cf**_0_), *I*_4s_ = **s**_0_ · (**Cs**_0_), and *I*_8fs_ = **f**_0_ · (**Cs**_0_), and the material axes **f**_0_, **s**_0_, and **n**_0_ characterise the myofibre direction, sheet direction, and the sheet normal in the reference configuration, respectively.

Following prior work,^30^ the first Piola-Kirchhoff stress for the skeleton is further modified as

(46) $${\mathbb{P}}^{e}=\frac{dW}{dF}-\frac{a}{2}\text{exp}\left\{b\left(I_{1}-3\right)\right\}F^{-T},$$

which will ensure that if F=I, ${\mathbb{P}}^{e}=0$. Parameters in (45)_1_ are chosen to be the same as in prior work,^32^ which were inferred by matching clinical measurement of left ventricular kinematics. These are *a* = 2.24 kPa, *a*_f_ = 2.42 kPa, *a*_s_ = 0.55 kPa, *a*_fs_ = 0.40 kPa, *b* = 1.62, *b*_f_ = 1.83, *b*_s_ = 0.77, and *b*_fs_ = 1.7. The value of the bulk modulus is reported in Table 1.

To examine the changes resulting from a different constitutive law and the addition of fibre structure in the same geometry, boundary conditions, and loading as in Chapelle et al,^8^ we first examine the case of a swelling cube. For this we consider three cases:

- myofibres are along the *x* direction with **f**_0_ = (1,0,0), **s**_0_ = (0,1,0), and **n**_0_ = (0,0,1);
- myofibres are along the *y* direction with **f**_0_ = (0,1,0), **s**_0_ = (0,0,1,), and **n**_0_ = (1,0,0) and
- myofibres are along the *z* direction with **f**_0_ = (0,0,1), **s**_0_ = (1,0,0), and **n**_0_ = (0,1,0).

The results of these simulations are illustrated in Figure 5, which can clearly show that the fibre-reinforced cube deforms very differently with different fibre structure and from the verification test in Figure 4. For example, in Figure 5 (A), the cube has expanded in both the *y* and *z*. This is caused by the stiffer material properties along the x-axis where the myofibres lie. Similar results are also seen in panels (B) and (C), where the cube swells in the cross-fibre directions.

### 4.3 | Perfusion in a dynamic left ventricle model

We now use the methods developed in the previous sections to investigate a more physiologically relevant case by preforming simulations in a previously developed left ventricle model.^32^ This particular LV geometry was reconstructed from a cardiac magnetic resonance (CMR) imaging study of a healthy volunteer. Details of the imaging protocols and reconstruction process, and development of a rule-based fibre architecture were provided previously.^32,33^

To account for the active tension, the first Piola-Kirckhoff stress for the skeleton is modelled as the sum of the active and passive stresses,

(47) $${\mathbb{P}}^{e}=\frac{dW}{dF}+{\mathbb{P}}^{a},$$

in which the active stress ${\mathbb{P}}^{a}$ is

(48) $${\mathbb{P}}^{a}=JT_{\text{a}}Ff_{0}⊗f_{0}\text{ with }T_{\text{a}}=F\left(t,{\text{Ca}}^{2+},\text{SL},T^{\text{req}}\right).$$

**FIGURE 6:**
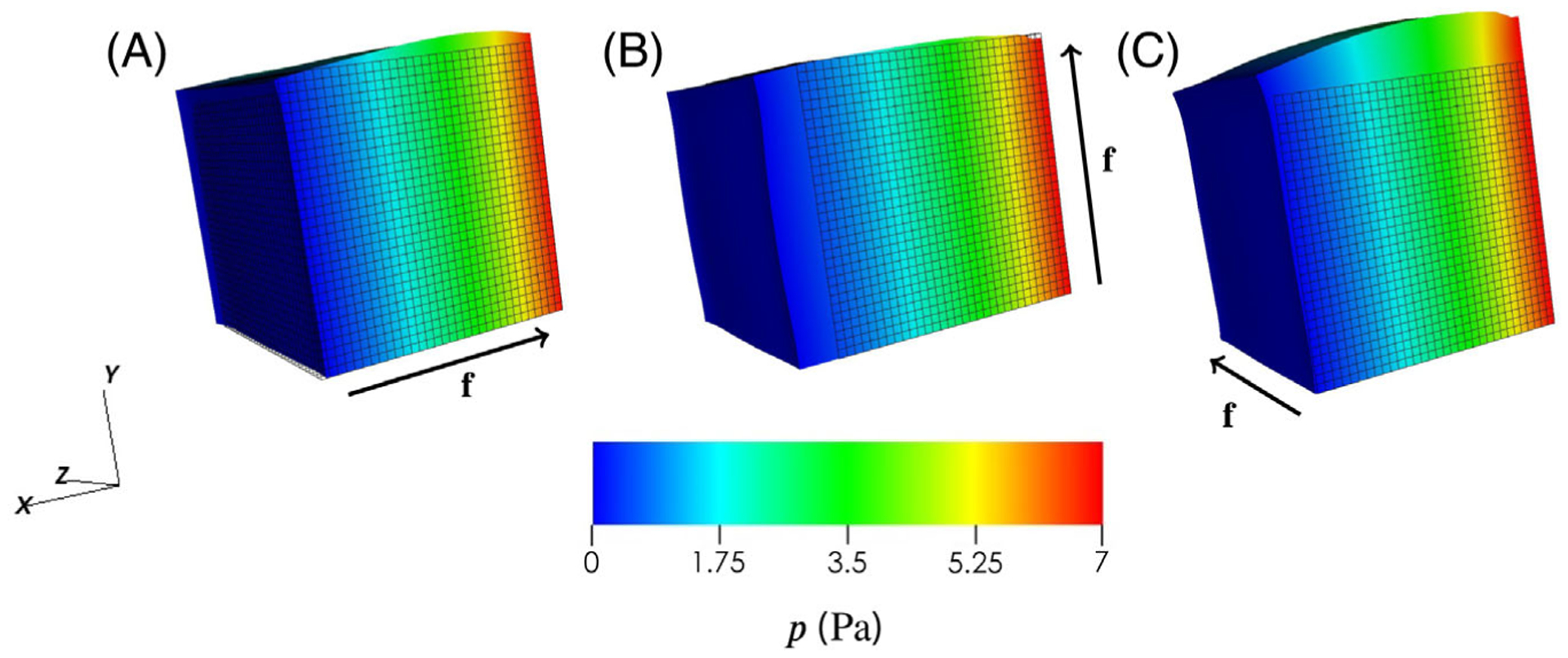
Simulation results when preforming the swelling test, as in Figure 4, but changing with the H-O material model (45). These results clearly show that altering the fibre direction changes the deformation with (A) illustrating fibres oriented along the *x* direction, (B) fibres oriented along the *y* direction and (C) fibres oriented along the *z* direction. For ease, the fibre directions are illustrated by the arrows alongside each result

In (48), *T*_a_ is the active tension determined by a group of ordinary differential equations F which depends on time, the intracellular calcium concentration Ca^2+^, sarcomere length (SL), and the required active tension *T*^req^ to achieve measured systolic volume, set at 124 kPa in this study. This active contraction model was detailed previously in Reference 34 (Figure 6).

As in Chapelle et al,^8^ we do not include multiple compartments, as it remains challenging to parameterize a real ventricular model in this way due to insufficient data on the distributions of coronary arteries, arterioles, capillaries, venules, and veins. Thus a single compartment describing a distributed source is used for the ventricular perfusion model. This is determined by

(49) $$S=\beta_{a}\left(p_{a}-p\right)-\beta_{v}\left(p-p_{v}\right),$$

in which *β*_*a*_, *β*_*v*_, *p*_*a*_, and *p*_*v*_ are parameters characterising the small coronary arteries and veins; see Table 1.

**FIGURE 7:**
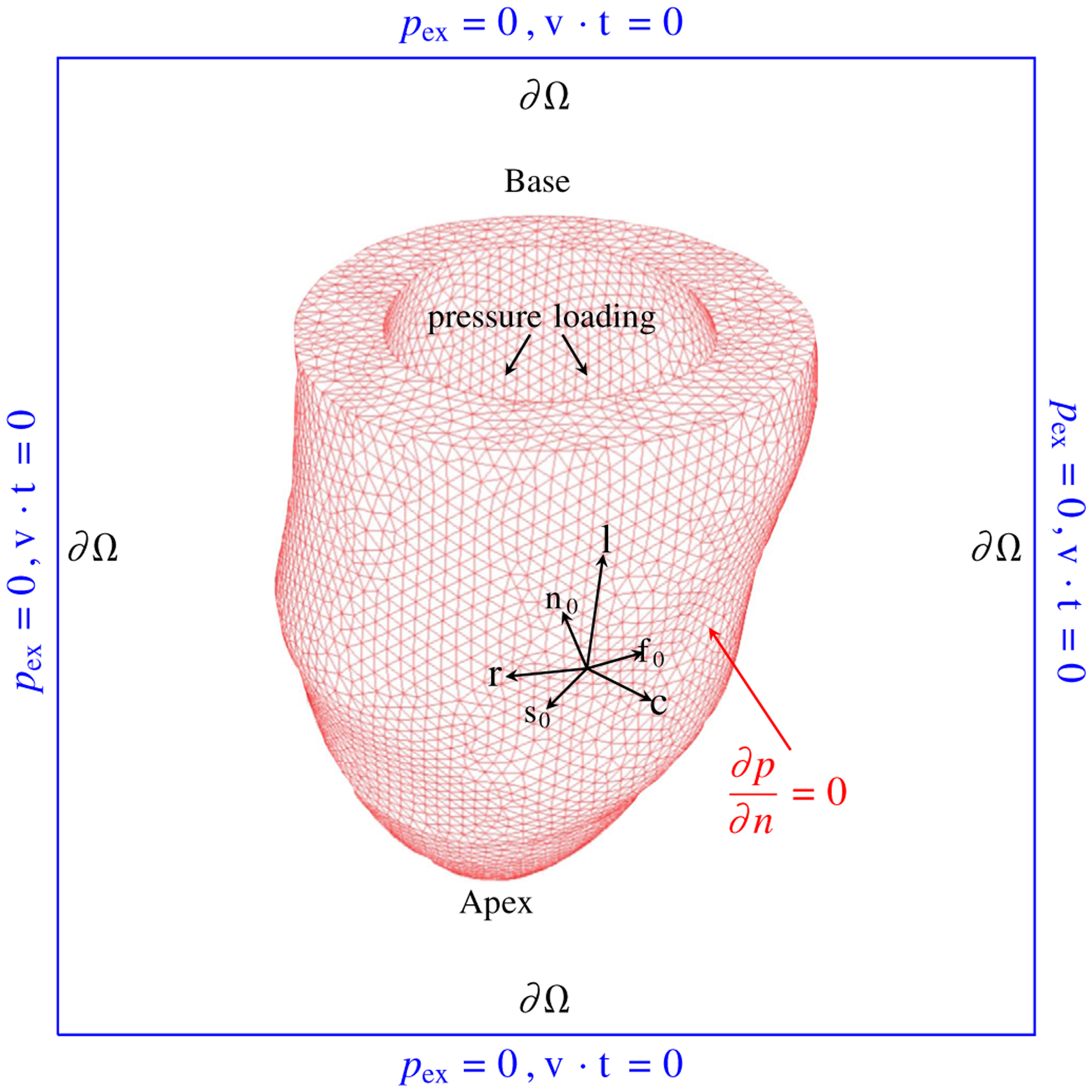
Illustration of the setting up of the LV model in the IB/FE poroelastic framework, adapted from Reference 30. **c**: the circumferential direction, **r**: the radial direction, **l**: the longitudinal direction. The blue box represents the fixed computational domain Ω with zero pressure and zero tangential slip along all boundaries, where **v** is the Eulerian velocity and **t** is the unit tangent vector

Figure 7 illustrates a schematic of the settings of the LV model within the IB/FE poroelastic framework. We set the properties of the bathing fluid to be blood, thus the LV cavity fluid (blood) will be merged with the bathing fluid. Note by including outflow/inflow tracts, the LV cavity fluid can be separated from the bathing fluid.^20^ An LV cavity pressure is applied to the endocardial surface directly, and the displacements of the basal plane are fixed in the circumferential and axial directions, while expansion is allowed radially. The pressure loading is applied by first linearly increasing the endocardial pressure to an assumed end-diastolic value (8 mmHg) without active contraction, denoted as the diastolic filling phase. Then the LV model enters into the systolic contraction phase in which the pressure rapidly increases to the end-systolic value (112 mmHg), estimated by the measured brachial arterial pressure when the CMR images were acquired The intracellular calcium concentration is simultaneously increased to its peak value to trigger the active tension generation and remains at its peak value afterwards. For the pore pressure, a no flux boundary condition is applied to the base, endocardial and epicardial surfaces. Instead, the blood moves in the out of the myocardium through the source term, see Equation (49). The initial porosity is set to *ϕ*_0_ = 0.15, and the permeability is assumed to be isotropic and homogeneous across the ventricular wall with a value of *k* = 2 × 10^−9^ Pa^−1^s^−1^. We emphasise that the passive strain energy function and active contraction model are different from those used by Chapelle et al.^8^ For comparison, we also simulate a purely hyperelatic LV model that does not include the porous flow in myocardium, corresponding to *ϕ*_0_ = 0. As in our previous studies,^32,34^ we simulate diastolic filling for the first 0.5 s and systolic ejection for the final 0.6 s, at which point a steady state is reached. In these simulations, we set Δ*t* = 1 ms and, as in previous simulations,^30^ use a 96 × 96 × 96 Cartesian grid. We also further compared the results for both the average pore pressure and maximum displacement at the end of diastole between a 96^3^ and a 112^3^ mesh with differences for these being in the region of 3% for the displacements and 3.5% for *p*. Although this convergence test is less detailed than that carried out for the cubic case, the convergence tests of the coupled LV model are similar to our previous study^30^ that a 96 × 96 × 96 Cartesian grid proved optimal for obtaining computationally efficient, accurate results for a hyper-elastic LV model.

**FIGURE 8:**
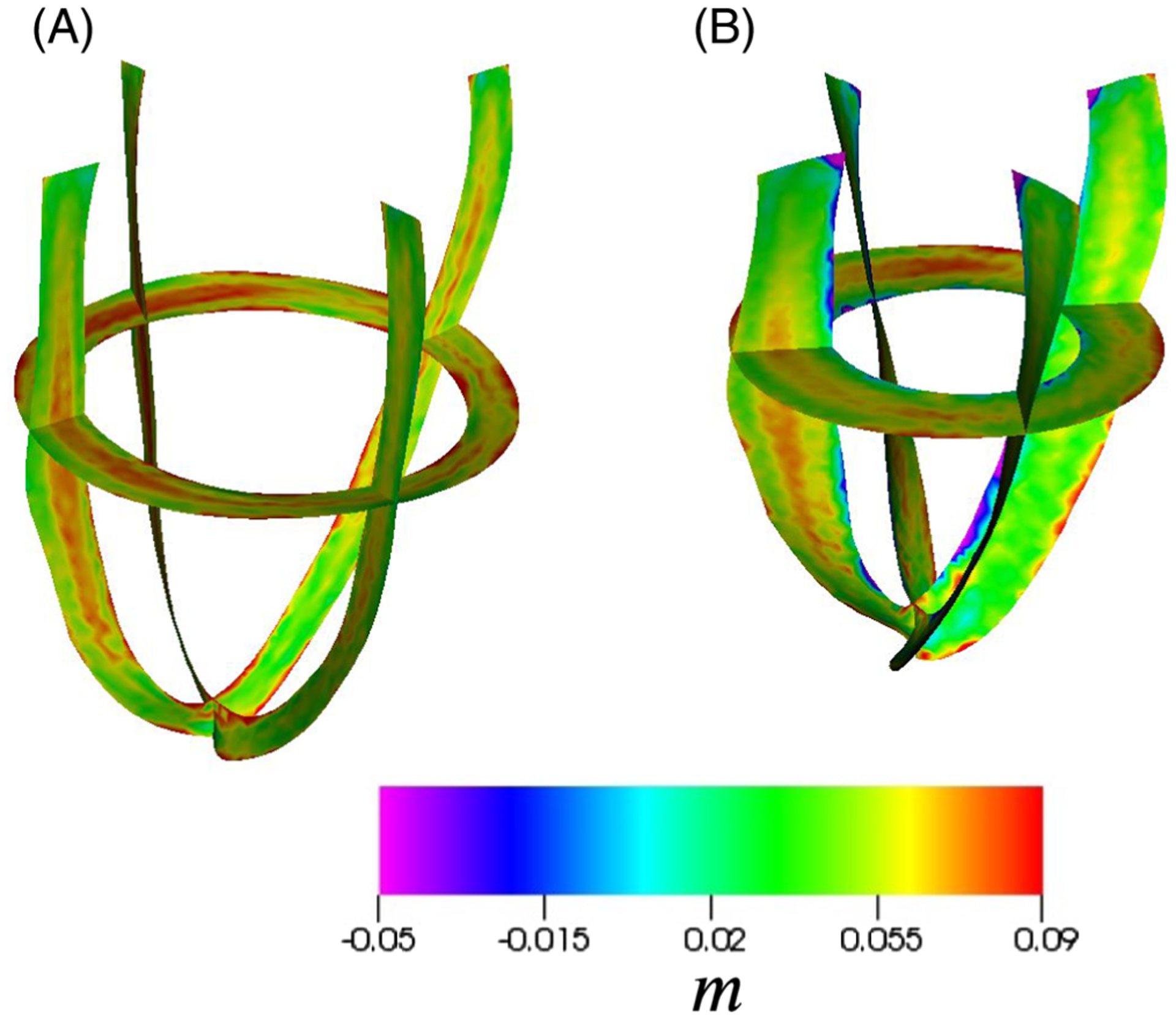
Simulation results of the LV model which are coloured by the added mass. The two images are plotted at different times with (A) corresponding to the end of dyastole and (B) the end of systole

Figure 8 shows the added blood mass to the myocardium at end-diastole and end-systole, respectively. During diastole, blood enters into the ventricular wall due to a lower pore pressure brought about by the passive expansion of ventricular wall, and the much higher coronary arterial pressure then pushes the blood into the myocardium with little flow out through the veins. In contrast, during systole, the active contraction exerts a much higher pore pressure in the myocardium, which pushes blood into the veins (represented by the negative values of added mass). Furthermore, as there is little inflow from the coronary arteries in systole, the net added mass drops below zero at this time.

**FIGURE 9:**
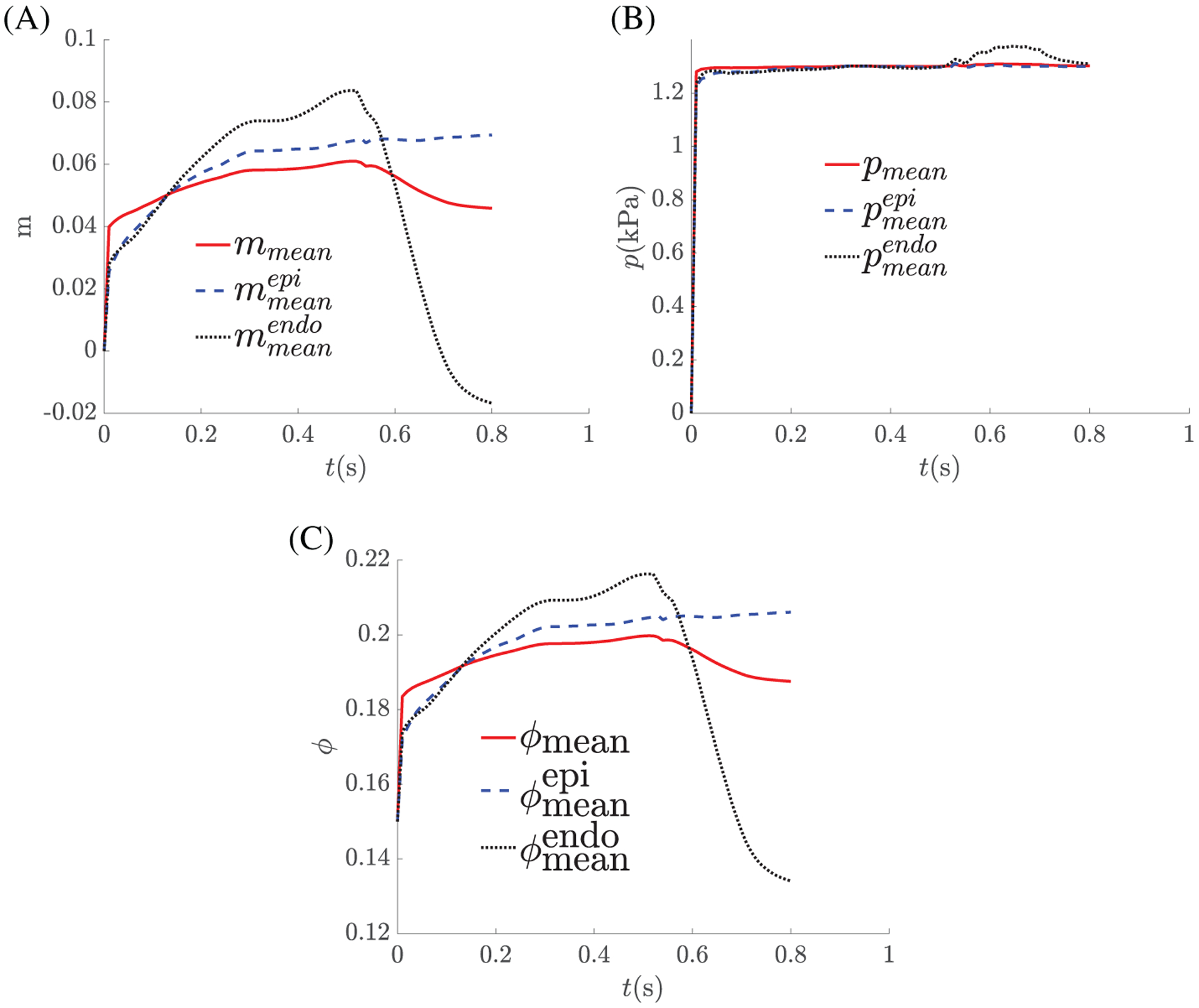
Time histories of the added mass, in terms of the wall-averaged value (*m*_mean_), epicardial surface averaged value ($m_{\text{mean}}^{\text{epi}}$), and endocardium surface averaged value ($m_{\text{mean}}^{\text{endo}}$), are plotted in (A), and the corresponding values of the pore pressure and porosity are shown in (B) and (C)

Figure 9 shows the time histories of the added mass in terms of the wall-averaged value (*m*_mean_), epicardial averaged value (*m*_epi_), and endocardial averaged value (*m*_endo_), along with the corresponding pore pressures. As expected, the averaged added mass increases during diastolic filling, although the local value depends on the transmural wall position, with a high value at the endocardium and a lower one at the epicardium. Similarly, during systole, the blood in the myocardium is actively squeezed out of the ventricular wall through the veins. This results in a higher value of *m* on the endocardium than that on the epicardium. This suggests that under our assumption of constant permeability, the perfusion inside myocardium would be transmurally heterogeneous, because the endocardium contracts more than epicardium. Figure 9(B) shows that the corresponding averaged pore pressures increase quickly at early diastole as the blood flows into the ventricular wall chiefly through the coronary arteries, since the coronary arterial pressure is much higher than the pore and venous pressures. Both *p*_mean_ and $p_{\text{mean}}^{\text{epi}}$ then remain more or less constant for the rest of cycle, presumably because most of the LV wall is well drained. Interestingly, there is a short rise in the endocardium pore pressure in systole, which seems to be associated with the sharp drop in the corresponding added mass at the same time.

**FIGURE 10:**
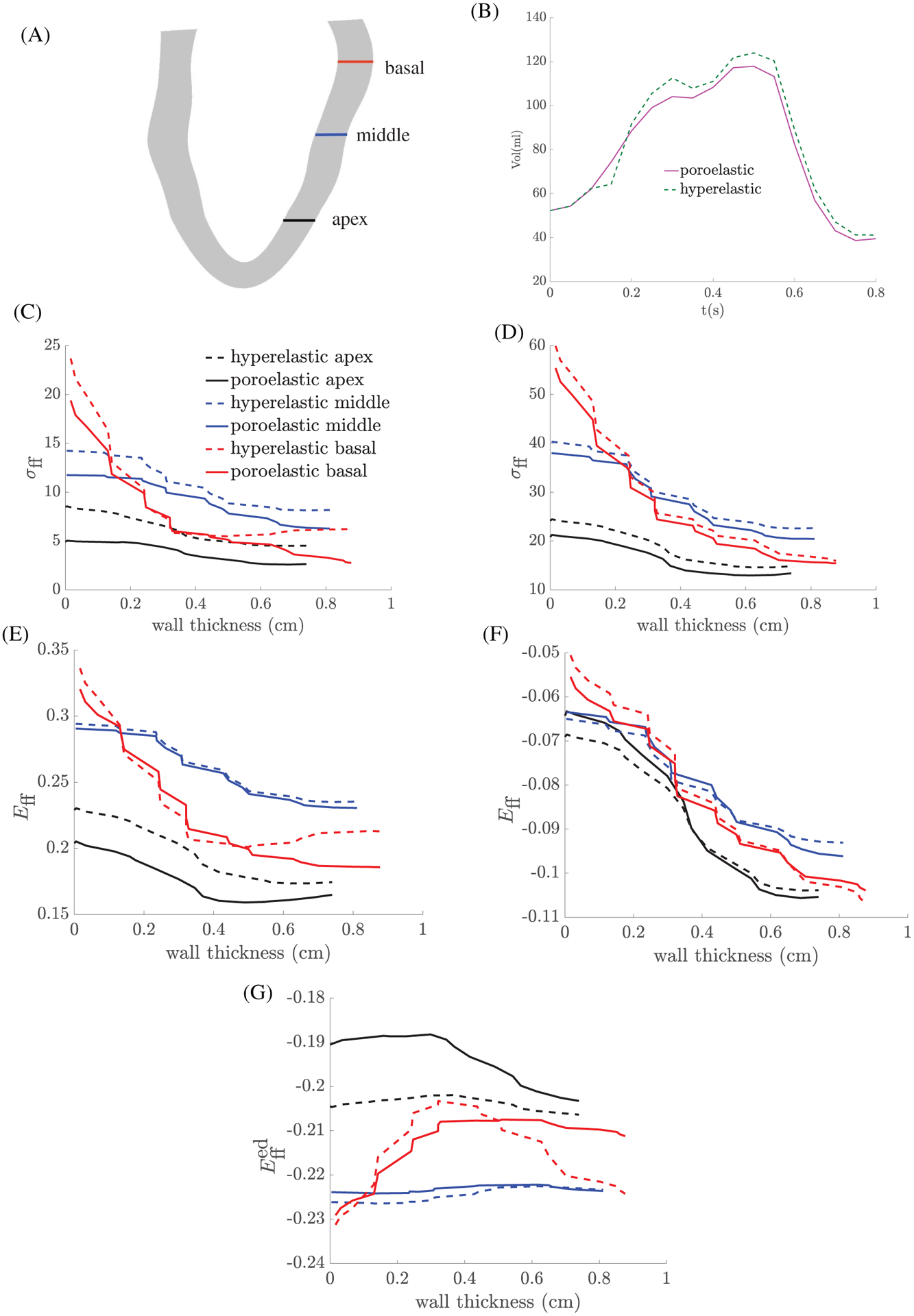
Comparison of the poroleastic (solid) and hyperelastic (dashed) models. (A) Three selected basal (red), middle (blue) and apex (black) locations, (B) volume history, (C, D) transmural (from endocardium to epicardium) fibre stress (indicated by *σ*_ff_) at the three locations as illustrated in (A), and (E, F) the corresponding fibre strain (indicated by *E*_ff_). The graphs in (C) and (E) are plotted at *t* = 0:5, corresponding to the end of diastolic filling, while (D) and (F) are at *t* = 0:8, approaching the end of systolic ejection. (G) as in (E) but for $E_{\text{ff}}^{\text{ed}}$, which is the fibre strain with respect to end-diastole, a strain measure commonly used in clinical studies^35^

Figure 10 compares the poroelastic and hyperelastic models. Figure 10(B) shows the ventricular volume history from diastole to systole for both poroelastic and hyperelastic LV models. Examining this, we see that for a given loading condition, the poroelastic model has a slightly lower filling volume in diastole with an end-diastolic volume of ≈117 ml compared to ≈124 ml in the hyperelastic LV model. This corresponds to a reduction of ≈7% and is in good agreement with the experimental findings from May-Newman et al,^36^ in which a 10% reduction in chamber volume was observed when comparing a regularly perfused heart to an unperfused one. Additionally, we note that this disparity is not as distinct during systolic contraction, where the respective volumes from the poroelastic and hyperelastic model are ≈39 ml and ≈41 ml. This is again consistent with the study from May-Newman et al,^36^ who suggested that systolic function is essentially unaltered by changes in perfusion.

Figure 10(E–G) compare the myofibre stress and strain transmurally at three different locations highlighted in Figure 10(A). We evaluate the fibre strain using two different methods, one being with respect to early diastole (the reference geometry), denoted as *E*_ff_, and the other with respect to end diastole, denoted as $E_{\text{ff}}^{\text{ed}}$. Although the former is more natural for computational modelling, the latter is more commonly used in clinical settings.^35,37^ Using both models, similar trends are seen in the stress and strain distributions. However, the differences are more pronounced in diastole than in systole, with lower fibre stress and strain in the poroelastic LV. This is most clearly seen in the diastolic distribution of $E_{\text{ff}}^{\text{ed}}$. The reduced strain and stress in diastole may be explained by the stiffening effect in diastole due to the increased fluid mass in myocardium. In systole, the same active tension is applied in both models. As this is much stronger than passive loading, it ultimately dominates and as such overwhelms any potential model differences in systole. However one exception occurs around a small region in the middle of the wall thickness along the basal section (indicated in red), where the fibre strain is slightly higher in the poroelastic model, which may result from the local geometry. We also note that the fibre stress and strain are very similar for the two models when in systole.

**FIGURE A1:**
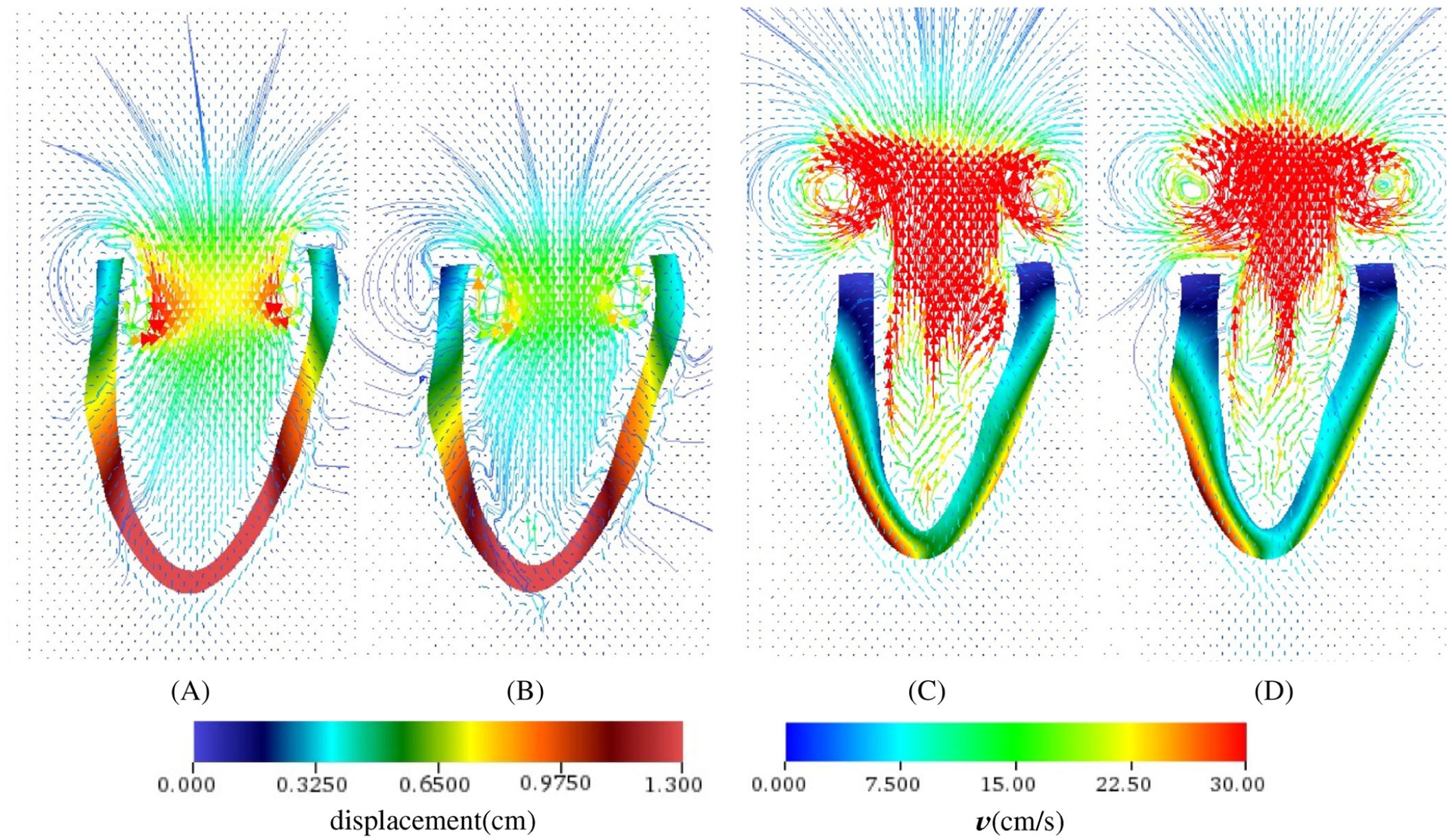
Cavity flow fluid and wall deformation of a longitudinal cross-sectional view of the (A) hyperelastic model, (B) poroelastic model in mid diastole (*t* = 0.25), and (C) hyperelastic model, (D) poroelastic model in mid systole (*t* = 0.7)

Comparisons of the cavity flow patterns and wall deformation between both models at mid-diastole and mid-systole can be found in the Appendix (Figure A1).

## 5 | DISCUSSION AND LIMITATIONS

The formulation of such models is in no small part possible due to various technological advancements enabling greater clinical capabilities. This has produced, for example, cardiac imaging equipment capable of generating highly detailed images of the coronary vessels which provide an essential template when developing the models which track blood flow through capillaries. However, the asymmetric and complex nature of this distributed vascular network, which moreover spans several orders of magnitude, reveal the significant challenges faced in accurately simulating the detail of this system, while the distinctive mechanical environment of periodic contractile cycles leads to a sustained fluid–solid interaction between coronary vessels and the myocardium in which they are embedded. Therefore, rather than considering the multitude of microscopic flows as unique, individual branches, within this study these are instead aggregated into a single macroscopic field perfusing the heart while the aforementioned fluid solid interaction is most effectively considered within the framework of poroelasticity. Previous models have indicated that these assumptions will not only produce physiologically accurate results but additionally, and of almost equal importance, they can provide a computationally effective solution. Using a simplified coronary network allows our model and subsequent methods to be benchmarked by comparing against a pair of test cases while the resulting formulation thereafter constitutes an established framework to be expanded upon making the next steps, of, for example, linking with a full vessel network, more easily achievable. Work to study heart perfusion to include detailed coronary network is on-going.

We also observe the expected stiffening effect in the poroelastic model compared to the unperfused hyperelastic model as shown in Figure 10, a reduction of 7% in end-diastolic volume. The experimental study by May-Newman et al^36^ found that by increasing perfusion pressure from 0 to 100 mmHg, a 10% reduction in LV chamber volume was observed, but this was accompanied by no changes in fibre strain They further suggested the systolic function may not be substantially impacted by changes in coronary perfusion. Our results show that the systolic function in the poroelastic model is very close to the hyperelastic model with slightly smaller systolic volume, and the ejection fractions generated by the two models are nearly same (66%). In fact, Arnold et al^38^ found that increased perfusion can even cause a positive inotropic effect. The same stiffening effect was also demonstrated in Cookson et al’s study^13^ with an isotropic material model for myocardium. As shown in Figure 10(C–F), large differences exist in the apical region, which may be partially explained by the geometry feature near the apex and the difficulty of defining realistic fibre structure at the apical region. Still, we find that the fibre strains in diastole for the two models at the basal and middle ventricle are very close, which is again consistent with the experimental findings from May-Newman et al.^36^ The contracting strains with respect to end-diastole are slightly smaller (less negative) in the poroelastic model overall, while we note that the values of strains from both models are well within the ranges reported in prior clinical studies.^35^

## 6 | CONCLUSION

This paper introduces a three-phase fluid–structure interaction model for simulating poroelastic structure immersed in a viscous incompressible fluid. Our formulation extends the hyperelastic immersed finite element approach of Griffith and Luo and through a simple benchmark tests, we obtained excellent agreement with prior results of Chapelle et al.^8^ In addition we found that the fibre structure of the myocardium has a substantial impact on the pore flow within the tissue. We then applied this approach to simulate ventricular mechanics using a human left ventricle model with an anisotropic hyperelastic elastic material model. Differences between the hyperelastic and poroelastic models are compared and discussed. We observe the expected myocardial stiffening in the poroelastic model caused by the perfusion from the coronary arteries in diastole, consistent with published experimental and numerical studies. While the systolic function from the poroelastic model is very close to a hyperelastic model with slightly smaller contracting fibre strain but nearly the same ejection fraction. Although this paper uses only a simple model of the coronary network, the new numerical framework developed paves the way for future more in-depth cardiac perfusion modelling.

## Figures and Tables

**TABLE 1:** Table of parameters

Name	Description/definition	Swelling test	Drainage test	Ventricle model	Units
*M*_*b*_	Biot modulus	2.18 × 10^5^	2.18 × 10^5^	2.18 × 10^5^	Pa
*b*	Parameter of the skeleton	1	1	1	—
*ρ*	Density	10^3^	10^3^	10^3^	kg m^−3^
*K*_*s*_	Bulk modulus for skeleton	2.2 × 10^5^	2.2 × 10^5^	2.2 × 10^5^	Pa
*κ*_0_	Penalty coefficient (as in^8^)	0.01	0.01	0.01	Pa
*ϕ*_0_	Initial porosity	0.1	0.1	0.15	—
**K**	Permeability tensor	$10^{-7}I$	$2.5\times 10^{-6}I$	$2\times 10^{-9}I$	m^2^ Pa^−1^ s^−1^
*β*_*a*_	—	—	—	3 × 10^−5^	Pa^−1^ s^−1^
*β*_*v*_	—	—	—	3 × 10^−2^	Pa^−1^ s^−1^
*p*_*a*_	—	—	—	2.7	kPa
*p*_*v*_	—	—	—	1.3	kPa

**TABLE 2:** Grid convergence tests using grid sizes of 64^3^, 80^3^, 96^3^, and 112^3^

*N*	Difference of *p* at the cube centre	Difference of the maximum displacement
64	≈10.5%	≈11.1%
80	≈4.8%	≈5.1%
96	≈3.8%	≈3.5%
112	≈3.7%	≈3.3%

*Note:* Relative differences are determined by comparing the results to those obtained using a 128^3^ grid.

## APPENDIX Numerical implementation of IB/FE poroelasticity

The numerical implementation of the pororelastic immersed finite element framework is shown in Algorithm 1.
